# Exploration of time sequential, patient specific 3D heart unlocks clinical understanding

**DOI:** 10.1186/s41205-018-0034-7

**Published:** 2018-12-06

**Authors:** Kylie A. Mena, Kevin P. Urbain, Kevin M. Fahey, Matthew T. Bramlet

**Affiliations:** 1 University of Illinois College of Medicine, 1 Illini Drive, Peoria, IL 61605 USA; 2 Jump Simulation, 1306 N. Berkeley Avenue, Peoria, IL 61603 USA; 3 OSF St. Francis Medical Center, 530 NE Glen Oak Avenue, Peoria, IL 61637 USA

**Keywords:** Congenital heart defects, Pediatric cardiology, Surgery, Patient-specific, 4D heart, Four-dimensional heart, Virtual reality

## Abstract

**Objectives:**

The purpose was to create a time sequential three-dimensional virtual reality model, also referred to as a four-dimensional model, to explore its possible benefit and clinical applications. We hypothesized that this novel solution allows for the visuospatial benefits of the 3D model and the dynamic benefits of other existing imaging modalities.

**Background:**

We have seen how 3D models hold great value in medical decision making by eliminating the variable visuospatial skills of practitioners. They have proved especially invaluable concerning the correction of congenital heart defects and have altered the course of many surgeries. There are, however, limitations to three-dimensional models. The static models only show what the heart looks like in one snapshot of its cycle and do not allow for an understanding of the physiological and dynamic processes.

**Methods:**

This solution segments a 3D heart derived from a 2D image stack, times the 18 phases of a cardiac cycle and creates a 4D model that can be manipulated in space and time through the use of virtual reality.

**Results:**

We believe the 4D heart provides a unique understanding of in situ cardiac anatomy not possible with other imaging techniques. Our expanding case series of clinician experiences and their immediate recognition of the potency of this technique is highly encouraging and reveals the future of functional and dynamic 4D representations of anatomy.

**Conclusions:**

The 4D heart improved our understanding around complex 3D relationships over time. We propose time and effort dedicated to 4D cardiac imaging analysis of dynamic cardiac pathologies such as hypertrophic obstructive cardiomyopathy or a pre-op Rastelli repair with a narrow outflow tract could offer tremendous insight into the medical decision-making process.

**Electronic supplementary material:**

The online version of this article (10.1186/s41205-018-0034-7) contains supplementary material, which is available to authorized users.

## Introduction

It has long been the standard to view medical pathologies on a two-dimensional plane, but the advent of creative technology allows for medical imaging to extend beyond the second dimension. Here we present what we call the four-dimensional heart - also referred to as a time sequential three-dimensional virtual reality model. We have seen in our lab how 3D models hold great value in medical decision making by eliminating the variable visual spatial skills of practitioners. They have proved especially invaluable in cases concerning the correction of congenital heart defects and have altered the course of many surgeries [1]. There are, however, limitations to three-dimensional models. The static models can only show what the heart looks like in one snapshot of its cycle and do not allow for an understanding of the physiological and dynamic processes. Additionally, the cost of the printer and material necessary to make the models may impede adoption of this practice [2]. There are other forms of imaging that add in a third or fourth dimension. The standard three-dimensional echocardiogram allows for a greater understanding than computerized tomography (CT) or magnetic resonance imaging (MRI), and multi-dimensional technologies such as the 4D flow MRI and the Computational Fluid Dynamics (CFD) computer model allow for in depth analyses of the physiology, but all are limited by their two-dimensional interactions on a screen [3]. The novel solution we devised was the 4D, time sequential 3D model that allows for the visuospatial benefits of the dynamic 3D model. As more CFD and 4D flow solutions enter the clinical field, this visualization technique will find more utility. This solution segments a 3D heart derived from a 2D image stack, times the 18 phases of a cardiac cycle and creates a 4D model that can be manipulated in space and time through the use of virtual reality. Our lab has worked with virtual reality technology in multiple areas of medicine including the cost-effective transition from printing physical 3D hearts to importing the 3D structure into a virtual reality platform with the same effect. We have also seen how a virtual reality platform originally developed within the University of Illinois as a communication device for surgical planning around virtual 3D models of congenital heart disease is now being used for medical education [4]. The efficiency and endless potential of this technology inspired us to create the 4D heart. We believe the 4D heart provides a unique understanding of in situ cardiac anatomy that is not possible with other imaging modalities.

## Methods

**Fig. 1:**
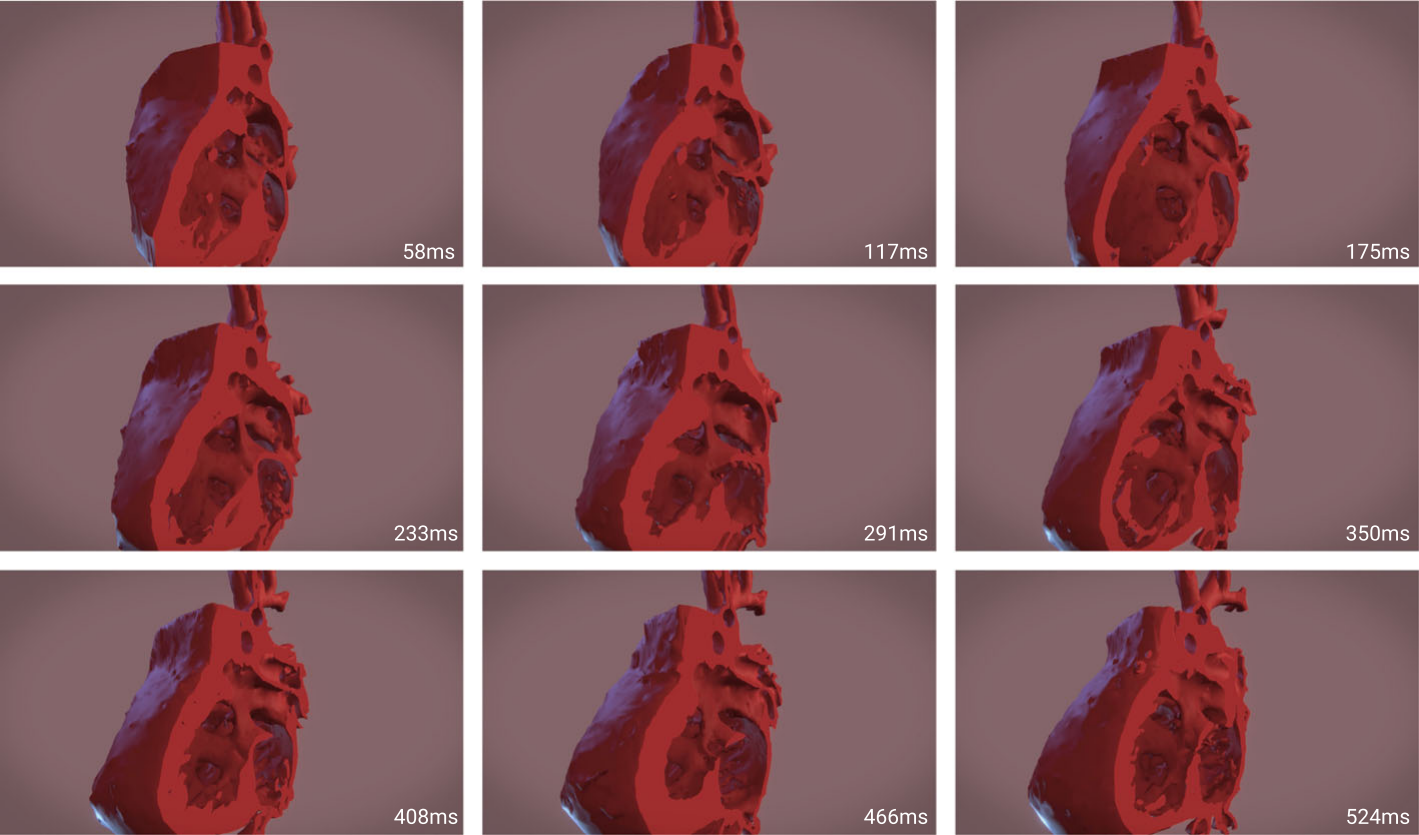
Image sequence from video showcasing motion of heart model with nine representative time points in cardiac cycle. Images above pulled every 7–10 frames from variable frame rate capture converted to 30fps

The steps in creating the 4D model were as follows: image acquisition, segmentation, quality control, animation, and virtual reality interaction. The images were collected using a GE revolution 256 slice scanner in axial mode at 0.625 mm. Kv =70. Smart mA range was 201–227 (smart MA mode 226) with rotation speed at 0.28 ms. They were captured from 5 to 95% of the cardiac cycle at 5% intervals. The images provided diagnostic quality data allowing for detailed tissue segmentation of myocardium, blood pool and vessel wall for each of the 18 phases of the cardiac cycle. A mask overlay of the myocardium was outlined on each slice of the 2D image stack for each phase of cardiac cycle. While blood pool segmentation of the contrast agent is fairly straightforward, myocardial segmentation is a more detailed, time consuming process of highlighting the vessel walls and myocardium [5]. An independent reviewer reviewed each segment to ensure that the myocardium and vessel walls were clearly represented for the key features of the case. Once each phase had a completed model they were imported into the Unity development environment in which a looping script toggled each model on and off in sequential order in rapid secession. The effect emulated a paper flip book, with each phase of the heart representing a different page in the book. The models with this attached code were imported into the Unity virtual reality platform producing the final 4D model (Fig. 1 and Additional file 1).

## Results and discussion

Once the 4D model was complete, there were observations made that we believe could not be visualized as clearly or at all using other methods. The first of these being that the sternum acts as the anchor for the cardiac motion (Additional file 2). Much of the current understanding of how the heart beats as an entire unit is tied to the three-dimensional perspective of seeing a heart beat in the operating room with an open chest. However, the native state of the heart beating within a closed chest is the opposite. The region of least resistance to expansion and contraction is at the base of the heart. This may seem intuitive, but seeing the heart beat in three-dimensions within the closed chest environment unlocks this understanding.

The relation of the tricuspid valve to the apex is much more obvious when viewed in the three-dimensional space with the perspective of the sternum acting as the anchor. This element is tracked in echo studies through the measurement of the tricuspid annular plane systolic excursion (TAPSE) distance as a surrogate of right ventricular ejection fraction. However, visualizing the tricuspid plane move in 4D allows for a better understanding of how conditions which affect the physical environment of the base of the heart can affect this value.

Additionally, the 4D model allows for a better understanding of how the papillary muscles move as the heart beats. When looking down through the mitral valve annulus, it can be appreciated that not only do the muscles contract, but they also move inward toward the center of the left ventricular cavity. This allows for a better understanding and appreciation of how this 4D movement correlates to the central coaptation of the mitral valve leaflets. Therefore, conditions that may alter this central movement of the papillary muscles, may actually contribute to mitral regurgitation.

Our expanding case series of clinician experiences and their immediate recognition of the potency of this technique is highly encouraging and reveals the future of functional and dynamic 4D representations of anatomy.

## Conclusions

A truth we have replicated continuously within our medical visualization laboratory is that we foster an improved clinical understanding when we translate patient-specific anatomy into virtual reality-mediated authentic representations of 3D structures. Like all explorers, we chose to create this 4D heart to see what we could discover. We believe that the translation of 2D medical images into 3D models represents a breakthrough innovation in medical imaging and that the translation of time sequential 3D cines into the 4D heart is an iterative advancement beyond the static 3D model generation.

It is rare to acquire any sequence where each of the 18 phases demonstrate high quality image resolution allowing for segmentation. Congenital cardiology will typically request cardiac MRI imaging over CT to minimize radiation doses, additionally, prospective CT gating techniques are preferred for the same reason. This case represented the rare combination where excellent images resulted from a retrospectively gated CT that was obtained due to the variable heart rate and hemodynamic instability of the patient. As more advanced radiation dose reduction techniques develop, we believe acquisitions of multiple phase cardiac imaging will become more frequent. The segmentation process for this case required a significant effort requiring approximately 2 to 3 h of segmentation per image set for each of the 18 phases of the cardiac cycle. Our lab is currently generating automatic segmentation software that would significantly decrease time spent on segmentation for future applications of this method [6]. We propose that time and effort dedicated to 4D cardiac imaging analysis of dynamic cardiac pathologies such as hypertrophic obstructive cardiomyopathy or a pre-op Rastelli repair with a narrow outflow tract could offer tremendous insight into the medical decision-making process.

## Additional files

Additional file 1: 2D video output of 4D heart as it rotates through cut plane. (MOV 51761 kb)

Additional file 2: Dr. Bramlet narrates findings within virtual environment while interacting with the model. (MP4 173683 kb)

## Abbreviations

2D: Two-dimensional
3D: Three-dimensional
4D: Four-dimensional
CFD: Computational fluid dynamics
CT: Computerized tomography
MRI: Magnetic resonance imaging
TAPSE: Tricuspid annular plane systolic excursion
